# Invasive non-typhoidal salmonella disease: an emerging and neglected tropical disease in Africa

**DOI:** 10.1016/S0140-6736(11)61752-2

**Published:** 2012-06-30

**Authors:** Nicholas A Feasey, Gordon Dougan, Robert A Kingsley, Robert S Heyderman, Melita A Gordon

**Affiliations:** a Malawi–Liverpool–Wellcome Trust Clinical Research Programme, Blantyre, Malawi; b Department of Gastroenterology, Institute of Translational Medicine, University of Liverpool, Liverpool, UK; c Wellcome Trust Sanger Institute, Hinxton, UK; d Liverpool School of Tropical Medicine, Liverpool, UK

## Abstract

Invasive strains of non-typhoidal salmonellae have emerged as a prominent cause of bloodstream infection in African adults and children, with an associated case fatality of 20–25%. The clinical presentation of invasive non-typhoidal salmonella disease in Africa is diverse: fever, hepatosplenomegaly, and respiratory symptoms are common, and features of enterocolitis are often absent. The most important risk factors are HIV infection in adults, and malaria, HIV, and malnutrition in children. A distinct genotype of *Salmonella enterica* var Typhimurium, ST313, has emerged as a new pathogenic clade in sub-Saharan Africa, and might have adapted to cause invasive disease in human beings. Multidrug-resistant ST313 has caused epidemics in several African countries, and has driven the use of expensive antimicrobial drugs in the poorest health services in the world. Studies of systemic cellular and humoral immune responses in adults infected with HIV have revealed key host immune defects contributing to invasive non-typhoidal salmonella disease. This emerging pathogen might therefore have adapted to occupy an ecological and immunological niche provided by HIV, malaria, and malnutrition in Africa. A good understanding of the epidemiology of this neglected disease will open new avenues for development and implementation of vaccine and public health strategies to prevent infections and interrupt transmission.

## Introduction

The bacterial genus *Salmonella* causes a huge global burden of morbidity and mortality. With regard to human disease, salmonellae are divided into typhoidal serotypes (*Salmonella enterica* var Typhi [*S* Typhi] and *Salmonella enterica* var Paratyphi A [*S* Paratyphi A]) and thousands of non-typhoidal salmonella serotypes (frequently referred to as NTS serotypes; panel). In high-income countries, non-typhoidal salmonellae predominantly cause a self-limiting diarrhoeal illness in healthy individuals; bloodstream or focal infection is rare^1^ and mainly happens in individuals with specific risk factors.^2^ By contrast, in sub-Saharan Africa, non-typhoidal salmonellae are consistently the most common bacterial bloodstream isolates in both adults and children presenting with fever,^3^, ^4^, ^5^, ^6^, ^7^ and are associated with a case fatality of 20–25%. Evidence from epidemiological studies; whole-genome sequencing of the pathogen; and discoveries about host immunity at the cellular, humoral, and mucosal level are helping to create a coherent picture of an emerging pathogen with a new pathogenesis. We will set these new findings in the context of what is known from other presentations of salmonella disease, and will discuss the similarities to and contrasts with typhoid fever.

Panel **Salmonella nomenclature**

The genus *Salmonella* consists of rod-shaped, Gram-negative, flagellated facultative anaerobes, and belongs to the family Enterobacteriaceae. Two species exist, *Salmonella enterica* and *Salmonella bongori*; all medically important salmonellae are included in the former, and are divided into more than 2500 serotypes on the basis of surface antigens, by use of a scheme initially devised by Kauffman. Serotypes have traditionally been named either for the syndrome they were thought to cause (eg, *Salmonella enterica* var Typhimurium [*S* Typhimurium] was thought to cause a typhoid-like syndrome in mice) or for the location of their discovery (eg, *Salmonella enterica* var Panama [*S* Panama]).

Salmonellae causing human disease are traditionally divided into a small number of human-restricted invasive typhoidal serotypes (eg, *Salmonella enterica* var Typhi [*S* Typhi] and *Salmonella enterica* var Paratyphi A [*S* Paratyphi A]) and thousands of non-typhoidal salmonella serotypes [commonly known as NTS serotypes]), which typically have a broad vertebrate host range and cause various presentations that usually include diarrhoeal disease.

## Historical perspective

Salmonellae were first identified in the late 19th century by the US Bureau of Animal Industry.^8^ Because a broad vertebrate host range exists, most salmonella diarrhoeal disease in developed countries is still zoonotic. Transmission is increasingly driven by commercial industrial production of meat, eggs, and processed food (eg, chocolate,^9^ jalapeño peppers,^10^ and peanut butter^11^).

Non-typhoidal salmonellae were described as a common cause of paediatric bloodstream infection in Africa in several reports that predate the HIV epidemic,^12^ and invasive non-typhoidal salmonellae were first noted in children with malaria in 1987.^13^ In 1983, shortly after AIDS was described, two cases of African adults with AIDS and *Salmonella enterica* var Typhimurium (*S* Typhimurium) bacteraemia were reported in Belgium.^14^, ^15^ In 1984, AIDS was identified in Africa (in Democratic Republic of the Congo and Rwanda), and these reports also included cases of invasive non-typhoidal salmonella disease.^16^, ^17^ The first case series of non-typhoidal salmonella bacteraemia was described in US patients with AIDS in 1984,^18^ and the first epidemiological link between invasive salmonella infection and AIDS was made in New Jersey.^19^ An age-stratified study in New York City then showed that invasive non-typhoidal salmonella infections were over-represented in registered patients with AIDS by a factor of 198, and in patients with multiple-site infections by a factor of 305.^20^ Recurrent non-typhoidal salmonella bacteraemia was added to the US Centers for Disease Control and Prevention case definition for AIDS in 1987. By 1990, non-typhoidal salmonella had been confirmed as a common HIV-related pathogen in sub-Saharan African adults.^6^

By contrast, the typhoidal serotypes *S* Typhi and *S* Paratyphi A are entirely host restricted to people and cause invasive disease in immunocompetent hosts. Although once a substantial public health problem in developed countries, improvements in sanitation have led to near eradication, and most cases are imported. A large burden of disease remains in developing countries, particularly in Asia. Typhoid fever (also known as enteric fever) is an invasive, systemic clinical syndrome that is typified by high fever and complicated by sepsis and shock, gastrointestinal bleeding or perforation, encephalopathy, and focal metastatic complications such as cholecystitis or hepatitis. The untreated case fatality is 20%,^21^ but is less than 1% with appropriate antimicrobial treatment.^22^ *S* Typhi persist in the reticuloendothelial system and biliary tract, from where bacteria are intermittently shed into the gastrointestinal tract, which leads to transmission of the disease to other people, potentially over many years. Persistence of *S* Typhi in the biliary tract is associated with an increased incidence of gallbladder carcinoma in endemic regions.^23^, ^24^

## Burden of disease and epidemiology

**Figure 1:**
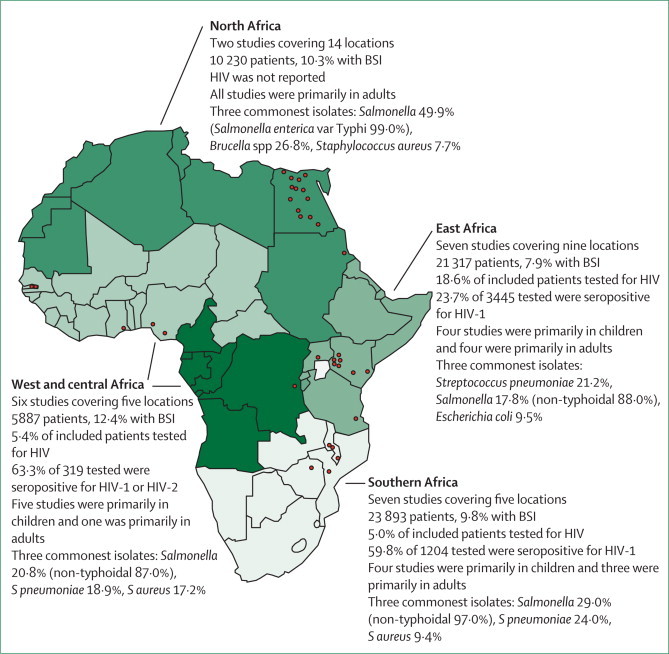
Map of Africa showing results of a meta-analysis of studies investigating the cause of bloodstream infection in febrile adults and children in Africa Reproduced from Reddy and colleagues.^3^ BSI=bloodstream infection.

Studies of bacteraemia have suggested that invasive non-typhoidal salmonellae are among the most common isolates from febrile presentations in adults and children across sub-Saharan Africa, especially where HIV prevalence is high (figure 1).^3^ The patchy availability of high-quality or affordable diagnostic microbiology facilities throughout Africa makes accurate documentation of the incidence of invasive non-typhoidal salmonella difficult.^25^ Underappreciation of disease burden because of inadequate diagnostics and reporting is common in a range of neglected tropical diseases.^26^, ^27^ The total burden of invasive disease attributable to invasive non-typhoidal salmonella in Africa has not been measured but is probably substantial, with an estimated annual incidence of 175–388 cases per 100 000 children aged 3–5 years,^7^, ^28^, ^29^ and 2000–7500 cases per 100 000 HIV-infected adults.^3^, ^4^, ^30^, ^31^ A pronounced bimodal age distribution of invasive non-typhoidal salmonella disease exists in Africa, in which children aged 6–36 months^32^, ^33^ and adults in their third or fourth decade are at greatest risk.^34^ Most cases of invasive non-typhoidal salmonella disease across Africa are due to either *S* Typhimurium or *Salmonella enterica* var Enteritidis (*S* Enteritidis),^3^, ^4^, ^13^ although investigators at some sites report contributions from other serotypes such as *Salmonella enterica* var Isangi (*S* Isangi) in South Africa,^35^ *Salmonella enterica* var Concord (*S* Concord) in Ethiopia,^36^ and *Salmonella enterica* var Stanleyville (*S* Stanleyville) and *Salmonella enterica* var Dublin (*S* Dublin)^37^ in Mali. Researchers at several sites have noted a decline in the incidence of invasive non-typhoidal salmonella disease, and some have noted a temporal association with decreases in malaria infections,^38^, ^39^ although this association is not universally reported (unpublished).

Although the incidence of invasive non-typhoidal salmonella disease is underestimated, the incidence of typhoid fever in Africa has perhaps been overestimated. However, few population-based data are available. A survey of the worldwide burden of typhoid estimated a crude incidence in Africa of 50 cases per 100 000 people per year,^40^ but this estimate was based on blood-culture data from vaccine trials^41^, ^42^ from Egypt and South Africa in the 1970s and 1980s and could have been inflated by outbreaks or pockets of disease. A review and a meta-analysis of blood-culture studies^3^, ^43^ suggest that the overall burden of *S* Typhi is lower than was previously estimated in sub-Saharan Africa. *S* Typhi is, however, the predominant invasive salmonella serotype in north Africa (where HIV is less prevalent),^3^ and important foci or outbreaks of *S* Typhi infection remain at some sites in sub-Saharan Africa.^44^, ^45^, ^46^ The investigators of two studies^3^, ^44^ have reported that HIV might be protective against typhoid fever.^3^, ^44^

By contrast with the findings in Africa, a multicentre fever surveillance report in Asia identified very little invasive non-typhoidal salmonella disease^47^ compared with the dominance of typhoid fever,^48^ although sampling of the youngest (and therefore most at risk) children was not done at some study sites. The explanation for such disparity between Africa and Asia is unclear, although the lower prevalences of *Plasmodium falciparum* malaria and HIV in Asia could be relevant. This pattern might change if HIV becomes more prevalent in Asia.^49^ Reports from developed countries in the pre-antiretroviral therapy era estimated annual incidences of non-typhoidal salmonella infections of roughly 400 cases per 100 000 patients with AIDS, greatly in excess of the rate in the general population.^20^, ^50^ However, some evidence shows that the incidence fell after the introduction of antiretroviral therapy.^50^

The contribution of non-typhoidal salmonellae to diarrhoeal illness in sub-Saharan Africa is poorly described and published work suggests a confusing situation. Culture-based studies of diarrhoea in sub-Saharan Africa have shown that non-typhoidal salmonellae are isolated in 2–27% of culture-positive diarrhoeal illness,^51^, ^52^, ^53^ but are also in the stools of 2–7% of asymptomatic controls. Thus, to attribute illness to stool positivity is difficult. A seroprevalence study of healthy children in Malawi revealed that they all had anti-*Salmonella* IgG antibodies by the age of 16 months, which suggests that infants have been universally exposed to either non-typhoidal salmonellae or cross-reactive antigens at a young age.^32^ A 2010 study of worldwide burden of non-typhoidal gastroenteritis estimated 2·5 million cases of the disease and 4100 deaths per year in Africa.^54^ However, these data were extrapolated from returning travellers, who are unlikely to be representative of rural or low-income Africans.

We do not know whether the same strains of non-typhoidal salmonella cause both invasive and diarrhoeal disease, or if not, whether the modes of transmission are the same. Basic questions about the environmental reservoirs and host ranges of invasive strains in Africa remain unanswered. Studies of food quality have identified non-typhoidal salmonellae in home-cooked food,^55^ fish from the great lakes of Africa, and market food,^56^ but typing beyond serotype or genus level was not done and the relevance to invasive disease is uncertain. An investigation into the households of index cases of paediatric invasive non-typhoidal salmonella disease in Kenya showed that 6·9% of human contacts carried non-typhoidal salmonellae in their stools, and 66% of isolates were similar to the invasive strain in the index patient by molecular analysis.^57^ By contrast, only unrelated strains of *S* Typhimurium, *S* Enteritidis*, Salmonella enterica* var Agona (*S* Agona), *Salmonella* enterica var Choleraesuis (*S* Choleraesuis), *Salmonella enterica* var Derby (*S* Derby)*,* and *Salmonella enterica* var Anatum (*S* Anatum) were isolated from livestock or the household environment, which raises the possibility that transmission is mainly between people. A comprehensive investigation into the epidemiology of invasive and non-invasive non-typhoidal salmonella strains in Africa is urgently needed,^25^ and data from the Global Enterics Multicentre Study could further clarify these issues in the near future.

Published accounts of invasive non-typhoidal salmonella in Africa show that the disease is highly seasonal.^4^ Peaks of infection during the rainy season in both adults and children coincide with increased incidences of malaria and malnutrition. Invasive non-typhoidal salmonella disease has also been present in epidemics that last several years and are caused by sequential single serotypes among adults and children. These epidemics have been linked to the emergence of resistance to commonly used antimicrobial drugs.^4^

## Clinical features in high-income countries

In high-income countries, non-typhoidal salmonellae mainly cause a self-limiting enterocolitis in immunocompetent individuals, in which patients present with nausea, vomiting, profuse watery diarrhoea, and abdominal pain.^58^ Up to 5% of patients will develop secondary bacteraemia,^58^, ^59^, ^60^, ^61^ but attributable mortality is probably low (1–5%). Non-typhoidal salmonellae can persist in the gastrointestinal tract after diarrhoeal illness, and this risk is increased by antimicrobial therapy.^62^ So-called primary non-typhoidal salmonella bacteraemia, without associated diarrhoea, occurs in at-risk groups—such as patients who are immunosuppressed because of HIV infection, steroid use, malignancy, chronic renal or liver disease, diabetes, or sickle-cell disease, and elderly and newborn patients.^2^ Mortality in adults was 12·2% in a hospitalised case series in Spain.^63^ Focal or metastatic disease also happens in patients with structural abnormalities, such as valvular heart disease, aneurysms or atherosclerosis, biliary or urinary tract abnormalities, bony abnormalities, or prostheses.^64^

Invasive non-typhoidal salmonella disease can be a complication of several inherited immunodeficiencies—eg, chronic granulomatous disease,^65^ in which phagocytes are unable to kill ingested organisms; sickle-cell disease, in which dysfunctional macrophages might cause susceptibility; and B-cell deficiencies.^66^ Notably, children with rare inherited deficiencies of components of the interleukin 12–interleukin 23 pathway have a particularly high incidence of recurrent invasive non-typhoidal salmonella infections,^67^ in keeping with the importance of these proinflammatory cytokines in the clearance of intracellular infections.

## Clinical features of invasive disease in sub-Saharan Africa

**Figure 2:**
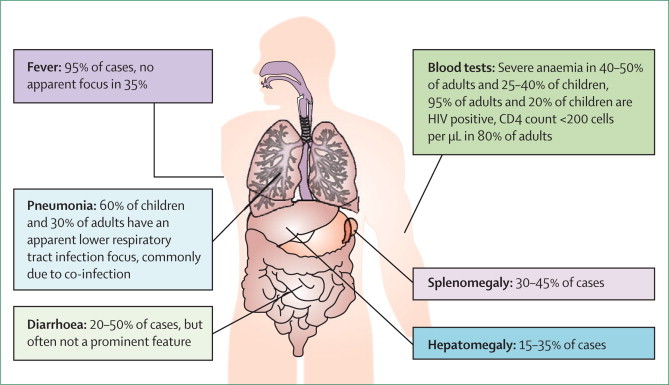
Clinical features of invasive non-typhoidal salmonella disease in adults and children in Africa

The clinical presentation of invasive non-typhoidal salmonella disease in Africa is typically febrile systemic illness^68^, ^69^, ^70^ resembling enteric fever; diarrhoea is often absent and other clinical features are diverse and non-specific (figure 2).^68^ A study of bacteraemia in adults in Malawi^69^ noted that a combination of high fever and splenomegaly suggested invasive disease, but that diagnosis cannot reliably be made without microbiological tests. In young children especially, a problematic clinical overlap exists with the presentations of pneumonia and malaria,^70^, ^71^, ^72^ and paediatric guidelines for empirical diagnosis and treatment in low-income settings fail to identify or treat invasive non-typhoidal salmonella disease. Patients with invasive disease frequently present with apparent focal infection, particularly of the lower respiratory tract, which is commonly attributable to co-infection with other pathogens such as *Mycobacterium tuberculosis*^73^ and *Streptococcus pneumoniae*.^68^ Even microbiologically confirmed invasive non-typhoidal salmonella disease treated with appropriate antimicrobial drugs has a case fatality of 22–47% in African adults and children.^4^, ^68^

Risk factors for invasive non-typhoidal salmonella disease in children include HIV infection; malnutrition;^70^ and malaria, especially severe malarial anaemia,^13^, ^74^ acute severe malaria,^75^ and recent malaria.^76^ Sickle-cell anaemia is also an important risk factor in African children.^77^ Invasive non-typhoidal salmonella predates HIV as an important clinical disease in children in Africa^13^, ^78^, ^79^, ^80^, ^81^ and, although HIV is independently associated with the illness in febrile paediatric admissions (odds ratio 2·6), only roughly 20% of African children who get non-typhoidal salmonella disease are infected with HIV.^4^, ^76^ The main risk factor in adults is undoubtedly advanced HIV infection. Case series typically show 95% of adult cases to be in people infected with HIV, of whom 80% have a CD4 T-lymphocyte count of fewer than 200 cells per μL.^68^ In adults infected with HIV, before the antiretroviral therapy era 20–40% of survivors had recurrence,^4^, ^68^ even after appropriate antimicrobial drugs; despite retreatment, up to 25% of patients had several recurrences. Genomic typing of index and recurrence strains suggests that recrudescence is more common than reinfection as a cause of recurrences.^82^

In addition to sepsis, invasive non-typhoidal salmonella can seed to the meninges, especially in children. In Malawi, *S* Typhimurium has been the second most common cause of bacterial meningitis since the introduction of the *Haemophilus influenzae* B vaccine, and accounted for 15% of culture-proven meningitis in 2008 (unpublished). The case fatality of salmonella meningitis is 52% in children and 80% in adults.^83^ Additionally, invasive non-typhoidal salmonella infection in African children is associated with active schistosomiasis,^84^, ^85^, ^86^, ^87^, ^88^ because the bacteria can adhere to the adult helminth's tegument,^89^, ^90^ where they can evade antimicrobial treatment. Treatment with praziquantel kills the adult helminth and allows antibacterial drugs to eradicate the bacteria. Schistosomiasis has not been shown to contribute to susceptibility, death, or recurrence of invasive non-typhoidal salmonella disease in adults infected with HIV.^91^, ^92^

## Management in Africa

Salmonellae were once susceptible to a broad range of affordable and effective antimicrobial drugs, but multidrug-resistant strains^4^, ^93^ have emerged in Africa. In Malawi, epidemics of multidrug-resistant invasive non-typhoidal salmonella (defined as resistant to ampicillin, chloramphenicol, and co-trimoxazole) have been recorded.^4^ *S* Typhimurium was shown to contain a composite element encoding multidrug-resistant genes located on a virulence-associated plasmid (pSLT-BT), thus potentially linking resistance to antimicrobial drugs with virulence.^93^ Resistance has necessitated the widespread use of expensive drugs for empirical management of sepsis, such as third generation cephalosporins and fluoroquinolones (eg, ciprofloxacin), which the poorest health systems in the world can ill afford, and which could promote the development of further resistance.

No intervention studies have been done to inform the best combined antimicrobial and antiretroviral therapy regimen to treat acute infection and prevent relapse. Infectious Disease Society of America guidelines for the management of invasive non-typhoidal salmonella disease in HIV-infected adults recommend 2–6 weeks of fluoroquinolone therapy.^94^ Fluoroquinolones, however, are also important in the management of drug-resistant tuberculosis, and since the two organisms can co-infect HIV-positive patients,^73^ further tuberculosis resistance could be promoted. Our experience is that rapid commencement of antiretroviral therapy can prevent relapse and therefore avert recurrent fluoroquinolone exposures (unpublished). Azithromycin is an attractive alternative antimicrobial drug. Ceftriaxone is probably an appropriate first-line intravenous treatment for patients unable to take oral drugs, but aminoglycosides do not penetrate intracellularly and are therefore not appropriate for treatment.

Recurrent invasive non-typhoidal salmonella disease, in the context of HIV, declines after antiretroviral therapy is started.^95^ The rollout of antiretroviral therapy will probably reduce the incidence of invasive illness in Africa.^50^

## Pathogenesis in immunocompetent hosts

**Figure 3:**
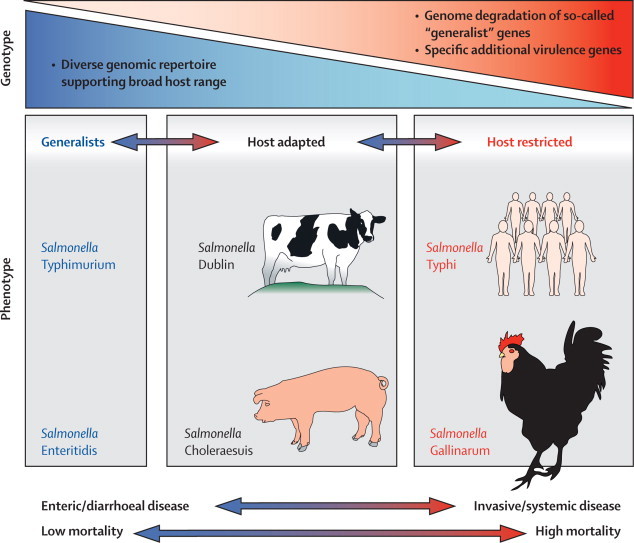
Features of host adaptation in salmonellae and effects on clinical syndrome in the host

In immunocompetent hosts, most clinical isolates of non-typhoidal salmonella cause inflammatory enterocolitis and diarrhoea, and can infect a broad range of potential vertebrate hosts. By contrast, typhoidal strains evade the gut mucosal immune response to cause systemic disease in a manner that is restricted to human beings and possibly analogous to the clinical picture noted in invasive non-typhoidal salmonella in Africa. Much progress has been made in understanding the mechanisms underlying the range of clinical syndromes reported, and their relevance to the host range and transmission of different serotypes (figure 3).

In relation to diarrhoeal disease, non-typhoidal salmonella can exploit the gut mucosal inflammatory response that accompanies infection in immunocompetent individuals to gain a selective advantage over the resident gut microbiota in the inflamed gut lumen.^96^ Many mechanisms probably underpin this mode of pathogenesis. For example most salmonellae harbour an *iro* gene cluster (*iroN, iroBCDE*), which can confer resistance to the host antimicrobial peptide lipocalin 2 by encoding a lipocalin-resistant siderophore that supplies iron in the inflamed gut.^97^ Salmonellae are also equipped to metabolically out-compete the microbiota in an inflammatory environment. For example, they can stimulate host-driven production of an electron acceptor that allows the pathogen to use respiration to compete with fermenting gut microbes.^98^ Thus, the symptoms of non-typhoidal salmonella diarrhoeal gastroenteritis are caused by inflammatory mechanisms that are also key to transmission of the luminal pathogen.

*S* Typhi, by contrast, generally avoid triggering a dominant primary gut mucosal inflammatory response through evasion mechanisms with virulence-associated adaptations such as the expression of the immunomodulatory Vi capsule and the accumulation of an inactivated gene repertoire that might favour entry through a non-inflammatory pathway. *S* Typhi also target small intestinal microfold cells, which are epithelial cells overlying Peyer's patches that sample the antigenic content of the gut.^99^ These cells direct *S* Typhi to underlying phagocytes such as dendritic cells and macrophages in the lamina propria, which favour intracellular dissemination to the lymphatics, the bloodstream, and viscera.^100^, ^101^ The bacteria persist in the reticuloendothelial system where they replicate before they are shed back into the circulation (precipitating symptoms of enteric fever) and into the gastrointestinal tract in infected bile. This alternative invasive strategy of typhoidal salmonellae ensures a long period of shedding by a small number of individuals and thus secures the onward transmission of this human-restricted organism.

## Invasive non-typhoidal salmonella disease in HIV-positive adults

**Figure 4:**
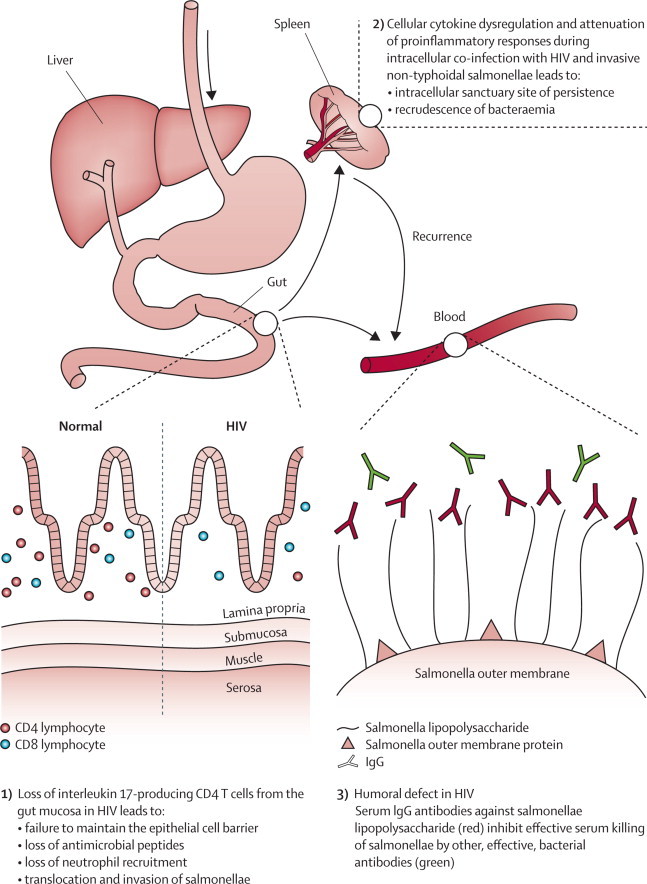
Three key defects that contribute to the pathogenesis of invasive non-typhoidal salmonellae in HIV-positive people Arrows show routes of dissemination of salmonellae.

Three key immunological defects have been described that could contribute to the invasive pathogenesis of non-typhoidal salmonella in HIV-infected adults in Africa (figure 4). First, an important defect has been identified in the gut mucosa. The gastrointestinal tract is a site of early and profound CD4 T-cell depletion in HIV infection,^102^ especially interleukin-17-producing T cells (Th17 cells). Th17 cells and their associated family of cytokines, including interleukins 17, 21, 22, and 26, coordinate mucosal defence through several mechanisms. They are crucial to the integrity, repair, and maintenance of the epithelial mucosal barrier, and induce epithelial cell expression of antimicrobial peptides such as β defensins and lipocalin. Additionally, Th17 cells have an important role in the stimulation of innate immune responses, by mediating neutrophil chemoattraction and function.^103^

A study in macaques positive for the simian immunodeficiency virus, which used a ligated ileal loop model, showed that infected animals had depleted interleukin-17-producing mucosal cells, blunted cellular Th17 responses, and reduced expression of genes implicated in the maintenance of the epithelial barrier.^104^ Bacterial translocation across the gut mucosa and systemic dissemination of salmonellae also took place. A key role for Th17 responses in protection against *Salmonella* dissemination was confirmed by use of a streptomycin-pretreated mouse model deficient in interleukin 17 receptors, which also showed reduced neutrophil influx and increased *S* Typhimurium invasion.^104^ These data show that loss of gut mucosal interleukin 17 cells in HIV infection is probably a key mechanism by which salmonellae disseminate from the gut to cause invasive disease in these patients. Loss of neutrophil chemoattraction could also explain the apparent absence of enteritis and diarrhoeal disease during HIV-associated invasive non-typhoidal salmonella.^68^

Second, dysregulated cytokine production during intracellular infection seems to allow persistence and recurrence of invasive non-typhoidal salmonella. Intracellular replication and persistence are key pathogenic competencies for salmonellae causing invasive disease. Quantitative blood cultures and bone-marrow cultures taken from HIV-positive adults with invasive non-typhoidal salmonella revealed a similar quantity of bacteria in both compartments at first presentation, but a six-times greater concentration of salmonellae in bone marrow compared with blood during relapse, which suggests persistence and replication. Furthermore, a substantial proportion of bacteria both in blood and bone marrow were in the intracellular compartment during invasive non-typhoidal salmonella infection. Bacterial load at relapse negatively correlated with cytokine concentrations and CD4 cell count, which suggests that failure of immunological control permitted escape from the intracellular compartment and symptomatic relapse.^105^

Investigation of intracellular killing and cytokine production by ex-vivo macrophages from HIV-infected adults did not show an intrinsic defect of macrophage killing, but instead demonstrated a highly dysregulated cytokine environment after *S* Typhimurium challenge. Tumour necrosis factor α, interleukin 10, and interleukin 12 were produced in excess during early HIV disease, but substantially reduced in late HIV disease, when adults infected with HIV are most susceptible to invasive non-typhoidal salmonella disease.^106^ Pronounced attenuation of proinflammatory cytokine responses is confirmed by transcriptional analysis of whole-blood responses.^107^ Cytokine responses are crucial for control of intracellular *Salmonella* infection,^108^ and the dysregulation and attenuation reported in HIV infection probably explain intracellular persistence of the bacteria, failure of immunological control, and episodes of relapse of invasive non-typhoidal salmonella disease recorded in advanced HIV infection.

Finally, the importance of antibodies both for serum killing and intracellular oxidative killing of invasive salmonella is increasingly recognised.^32^, ^108^, ^109^ A new humoral defect has been described in Malawian adults infected with HIV, a proportion of whom had impaired serum killing of non-typhoidal salmonella strains. Paradoxically, this impairment was associated with the presence, rather than absence of IgG antibodies directed against *Salmonella*. The antibodies, directed against *Salmonella* lipopolysaccharide, were shown to impair serum killing by blocking or competing with co-existing effective bactericidal antibodies directed against *Salmonella* outer membrane proteins.^110^ Although the clinical importance of these antibodies is still not proven in terms of a relation with disease susceptibility or outcomes in patients, this finding could have some implications for the choice of targets for potential vaccines.

## Immunological defects in African children

In children, other disorders are more important risk factors for invasive non-typhoidal salmonella than is HIV infection, especially severe malarial anaemia and malnutrition. The mechanism through which malaria predisposed to invasive non-typhoidal salmonella was thought to be mainly macrophage dysfunction as a result of iron release after haemolysis, but work in mice suggests a more complex interplay between the bacteria and malaria that involves both cytokine dysregulation^111^ and haem oxygenase-dependent dysfunctional granulocyte mobilisation.^112^ Although homozygosity for sickle-cell anaemia is a risk factor for invasive non-typhoidal salmonella,^77^ heterozygosity is protective against bacteraemias, probably because of the strong protection afforded against malaria.^38^ By contrast with the situation described in HIV-infected adults, African children aged 4–16 months have poor serum killing of *Salmonella* associated with an early deficiency of anti-*Salmonella* IgG and an excess of invasive non-typhoidal salmonella.^32^ Furthermore, invasive non-typhoidal salmonella infection is less common in the first 3–4 months of life than in later childhood, consistent with temporary protection afforded by transplacental protective antibodies, antibodies in maternal colostrum, or avoidance of environmental exposure during exclusive breastfeeding. Together, these findings suggest that antibodies play an important part in protection against invasive non-typhoidal salmonella in children.

## Evolution and host adaptation of *S* Typhimurium in Africa

To understand the pathogenesis of invasive non-typhoidal salmonella disease, both human-host immunity and the substantial genetic diversity of the genus *Salmonella* must be considered. Host-restricted salmonellae such as *S* Typhi, which are traditionally associated with systemic disease, have evolved a more restricted genomic repertoire, whereas salmonellae that maintain a broad host range predominantly cause enteritis (figure 3). The loss of functional gene capacity in invasive serotypes such as *S* Typhi and *S* Paratyphi A is at least partly attributable to the inactivation of genes that allow persistence of promiscuous serotypes in the intestinal lumen and the ability to target and manipulate host cells.^113^, ^114^ Loss of functional gene capacity with genome degradation from a progenitor with a broad host range has been reported in other human-restricted pathogens such as *Mycobacterium leprae*,^115^ *Yersinia pestis*,^116^ *Bordetella pertussis*,^117^ and *Rickettsia* spp.^118^

**Figure 5:**
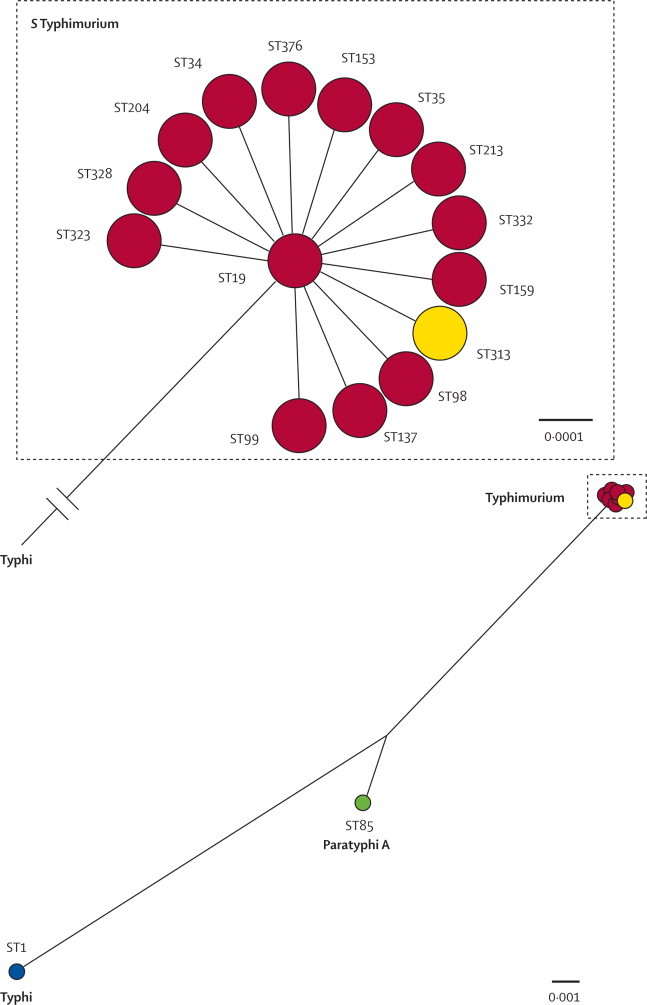
Radial phylogram showing the phylogenetic relations of *Salmonella enterica* var Typhimurium (*S* Typhimurium) sequence types (STs) The *S* Typhimurium sequence types (red or yellow circles) are rooted to *Salmonella enterica* var Typhi (*S* Typhi) (blue circle) and *Salmonella enterica* var Paratyphi A (*S* Paratyphi A) (green circle). Genetic distance is calculated as the number of single nucleotide polymorphisms per base and is indicated as a solid black line. The length of this line is proportional to the degree of genetic divergence. *S* Typhimurium sequence types from various worldwide locations outside sub-Saharan Africa (red) and from sub-Saharan AfrIca (yellow) are shown.

The *Salmonella* serotypes that most commonly cause invasive non-typhoidal salmonella in Africa are *S* Typhimurium and *S* Enteritidis, which are usually associated with a broad host range and with enteric disease.^119^ Whole-genome sequencing and PCR analysis done on a series of invasive isolates of *S* Typhimurium from Malawi and Kenya identified a dominant regional genotype—multilocus sequence type (ST) 313, which is uniquely found in Africa, and which has several genetic differences compared with other strains of this serotype (figure 5).^93^ Although other genotypes of *S* Typhimurium such as ST19 (which includes classic gastrointestinal strains such as DT104) can also cause invasive non-typhoidal salmonella in Africa, ST313 isolates are dominant in many sub-Saharan regions. ST313-like isolates from central Africa have been retrospectively identified as early as the 1980s (Al-Mashhadani M, Parry CM, University of Liverpool, personal communication).

An important characteristic of ST313 isolates is a degraded genome capacity in the form of pseudogenes and deletions. 60% of this degraded genomic repertoire is also degraded in *S* Typhi and *S* Paratyphi. Some of these genes have known functions relating to pathogenesis and some are of unknown function, but the convergence between ST313 and *S* Typhi*,* despite the distinct phylogenetic lineages of these *Salmonella* species^120^ (figure 5) raises the possibility that ST313 has adapted to occupy a unique niche in Africa and undergone convergent microevolution to become human adapted.^93^

We do not know whether these ST313 strains cause an appreciable burden of gastroenteritis in Africa. However, *Salmonella* isolates of an identical type were recovered from healthy people cohabiting with index cases of invasive non-typhoidal salmonella disease in Kenya, which suggests that asymptomatic carriage could be important in the transmission of these pathogens.^57^ Molecular epidemiological analysis of invasive and diarrhoeal strains of *S* Typhimurium and *S* Enteritidis will be valuable to understand and interrupt the transmission of invasive ST313 strains in Africa. However, the immunological and genetic characteristics of the host could still be the dominant factor affecting susceptibility to invasive non-typhoidal salmonella.

## Future research priorities

The true burden of salmonella disease in Africa is unclear. A comprehensive epidemiological study of invasive non-typhoidal salmonella, diarrhoeal non-typhoidal salmonella, and *S* Typhi that assesses the possible methods of transmission is urgently needed. The potential value of whole-genome-sequencing technologies has already been shown in epidemiological investigations of *S* Typhi in Asia.^121^ *S* Typhimurium ST313 might be becoming human adapted, and investigation of the reservoirs and risk factors for exposure and transmission will provide hypothesis-driven approaches to preventive measures—eg, vaccines, improved hygiene and sanitation, improved nutrition, malaria control, and antiretroviral therapy programmes.

Clinical algorithms fail to reliably identify invasive non-typhoidal salmonella in adults or children in Africa. Blood culture and microbiological diagnosis of *Salmonella* requires technical expertise and investment in infrastructure, consumables, and quality control, all of which must be strengthened in the region. An urgent need exists for the development of rapid, accurate point-of-care diagnostics to supplement conventional diagnostic microbiology. Targets for a non-typhoidal salmonella rapid multiplexed PCR diagnostic have been identified,^37^ but low numbers of the bacilli in blood make this process a technical challenge.^104^ Similar limitations have been noted in typhoid fever.^122^ A new ultra-fast method for detecting the *Salmonella ori C* chromosomal locus shows promise but needs validation and translation to real-world application.^123^

Vaccine development is a potential prospect for non-typhoidal salmonella control. The few *Salmonella enterica* vaccines so far have targeted *S* Typhi in people, but several approaches could be used to vaccinate against invasive disease. Inactivated whole cells have historically provided the basis for oral vaccines, such as those for cholera.^124^ This approach would be cheap, but its effectiveness against invasive disease is unclear. Live oral African non-typhoidal salmonella vaccine strains that were attenuated by mutations in guanine synthesis and flagellum regulatory genes protected mice against lethal infection with both *S* Typhimurium and *S* Enteritidis—such strains could be used as part of a vaccination strategy.^125^ A second approach, pioneered with the Vi antigen of *S* Typhi,^126^ would be to target surface polysaccharides (particularly O sidechains) in the form of a conjugate vaccine, and some programmes are pursuing this strategy. Different non-typhoidal salmonella serotypes, however, have immunologically distinct O sidechains, and consequently a multiple-antigen vaccine might be needed. A further approach could target surface protein antigens such as the porins or flagella.^125^, ^127^, ^128^ Protein antigens can be protective in mice, but this effect has not yet been translated into human vaccines. Non-typhoidal vaccine development is also complicated by the immunocompromised nature of susceptible patients,^108^, ^110^ which should be addressed early in clinical development.

## Search strategy and selection criteria

We searched Medline, PubMed, Embase, and the Cochrane Library to identify studies published between 2000 and 2011 about risk factors for, and epidemiology, pathogenesis, and treatment of, invasive salmonellae. Our search terms were “salmonella” in combination with “typhi”, “nontyphoidal”, or “Africa”. We did not limit our search by language. We mainly selected articles published in the past 10 years, but did not exclude important older reports. We also searched the reference lists of articles identified by this search strategy. Selection criteria included a judgment about importance of studies and their relevance for the well informed general clinician. We cite review articles where they provided comprehensive overviews beyond the scope of our Review.
